# Zinc- and magnesium-doped hydroxyapatite-urea nanohybrids enhance wheat growth and nitrogen uptake

**DOI:** 10.1038/s41598-022-20772-w

**Published:** 2022-11-14

**Authors:** Bhaskar Sharma, Luis O. B. Afonso, Manoj Pratap Singh, Udit Soni, David M. Cahill

**Affiliations:** 1 School of Life and Environmental Sciences, Deakin University, Geelong Waurn Ponds Campus, Geelong, VIC 3216 Australia; 2 Department of Botany and Plant Sciences, University of California-Riverside, Riverside, CA 92521 USA; 3 Department of Biotechnology, TERI School of Advanced Studies, New Delhi, 110070 India; 4 AIRF, Jawaharlal Nehru University, New Delhi, India

**Keywords:** Plant sciences, Environmental sciences

## Abstract

The ongoing and unrestrained application of nitrogen fertilizer to agricultural lands has been directly linked to climate change and reductions in biodiversity. The agricultural sector needs a technological upgrade to adopt sustainable methods for maintaining high yield. We report synthesis of zinc and magnesium doped and undoped hydroxyapatite nanoparticles, and their urea nanohybrids, to sustainably deliver nitrogen to wheat. The urea nanohybrids loaded with up to 42% nitrogen were used as a new source of nitrogen and compared with a conventional urea-based fertilizer for efficient and sufficient nitrogen delivery to pot-grown wheat. Doping with zinc and magnesium manipulated the hydroxyapatite crystallinity for smaller size and higher nitrogen loading capacity. Interestingly, 50% and 25% doses of urea nanohybrids significantly boosted the wheat growth and yield compared with 100% doses of urea fertilizer. In addition, the nutritional elements uptake and grain protein and phospholipid levels were significantly enhanced in wheat treated with nanohybrids. These results demonstrate the potential of the multi-nutrient complexes, the zinc and magnesium doped and undoped hydroxyapatite-urea nanoparticles, as nitrogen delivery agents that reduce nitrogen inputs by at least 50% while maintaining wheat plant growth and nitrogen uptake to the same level as full-dose urea treatments.

## Introduction

The world is facing a food crisis as the global population continues to rise, and current agricultural production will not be sufficient to meet future food demands^1^. It has recently been estimated that a 119% increase in edible crop production will be required to feed the, approximately, 9.7 billion people worldwide^2^. Along with the population increase, the use of fertilizers in agriculture over the last five decades has dramatically increased to meet the desired agricultural production^1,3^. A large amount of chemical fertilizer is routinely applied to agricultural land for the maintenance of crop yield, but this extensive use has serious environmental implications^3,4^. Application of a high amount of synthetic fertilizers is thought to promote crop yield over shorter periods^5^. However, plants require only 15–30% of the applied fertilizer, and the remaining chemical contaminates water bodies and soil^6^.

The global food system and agricultural industries that support it affect the environment through impacts on the soil, air and water, increased land use, biodiversity loss and the production of greenhouse gas emissions^2,3^. Chemically synthesized fertilizers, for example, contain residues of acids such as sulfuric acid and hydrochloric acid, which dissolve soil crumbs and negatively affect soil health. The prolonged use of chemical fertilizers alters nutrient content in the soil^7^, diminishes the entry of rainwater into the soil by altering porosity^8,9^, induces pest attack^10^, reduces soil water holding capacity^9^, soil aggregation and friability^11^, and upsets soil pH^8^, resulting in eradication of beneficial microorganisms from soil that then affects plant immunity and soil nutrient availability^12,13^. Higher fertilizer inputs influence plant–microbe interactions, and the fast-release chemical fertilizers with high nitrogen content induce numerous bacterial and fungal diseases in plants^10,14,15^. These induced effects ultimately lead to cycles of increased pesticide use those impacts both living organisms and their environment. Therefore, it is necessary to find new ways to use fertilizers that reduce synthetic fertilizer input into the soil while maintaining agricultural output^16,17^. Nitrogen is a crucial component of chemical fertilizers and can be a significant cause of environmental pollution^18^. If nitrogen runoff from agricultural fields can be diminished through targeted nutrient delivery to the plants, for example, by the incorporation into nanomaterials, then nitrogen administration can be reduced^19^.

Several nanomaterials have been utilized as plant growth stimulants, nano biosensors, and for the delivery of nutrients, pesticides, hormones and genetic material to plants^20^. However, the chemical or physical nature of nanomaterials may limit biological or environmental applications. For instance, heavy metals, transition metals, and carbon materials at the nanoscale exhibit excellent and flexible electronic, optical, and structural properties, however, their use in agriculture or the broader environment may be avoided due to the potential for off-target impacts^21,22^. Therefore, the selection and fabrication of nanomaterials needs to be very carefully considered to be suitable for large-scale agricultural applications.

Alternatives to conventional fertilizers are eagerly sought and include emerging alternatives such as slow-release fertilizers^23,24^, implementation of organic farming methods^25–27^, nutrient foliar sprays^28^, and nano-fertilizers^29,30^. Nano-fertilizers are a subset of nano-scale dimension particles that exhibit unique properties such as high surface area, surface charge, smaller size, and various shapes^31,32^. These properties can be utilized to integrate nutrient molecules with nanoparticles for controlled release and prolonged nutrient availability in the soil, thus, minimizing chemical fertilizer application and precise nutrient delivery to plants^33–35^. Nanoparticles can be administered either as nutrient molecules themselves or loaded with nutrient molecules for targeted delivery^30^. Nanoscale molecules have higher surface area, smaller size, and flexible surface chemistry that offer seamless engineering and fabrication opportunities for designing effective nutrient delivery systems^29,36,37^.

Hydroxyapatite is a biodegradable, highly adsorbent, nutrient-rich, and naturally available material at a lower cost that can be engineered to deliver nitrogen nutrients to the plants^37,38^. The hydroxyapatite-based nano-fertilizers slowly release nitrogen into the soil environment and stay in the soil for a longer time, thereby reducing nitrogen inputs to the soil^38^. In this study, the multi-nutrient complexes, urea-coated hydroxyapatite, urea-coated magnesium doped hydroxyapatite, and urea-coated zinc doped hydroxyapatite nanohybrids were synthesized, characterized, and used as a nitrogen nano-fertilizer for wheat plants. The nanohybrid characteristics, the release of nitrogen, and nitrogen delivery were examined across the life cycle of wheat.

## Results

### Nanomaterial characterization

**Figure 1:**
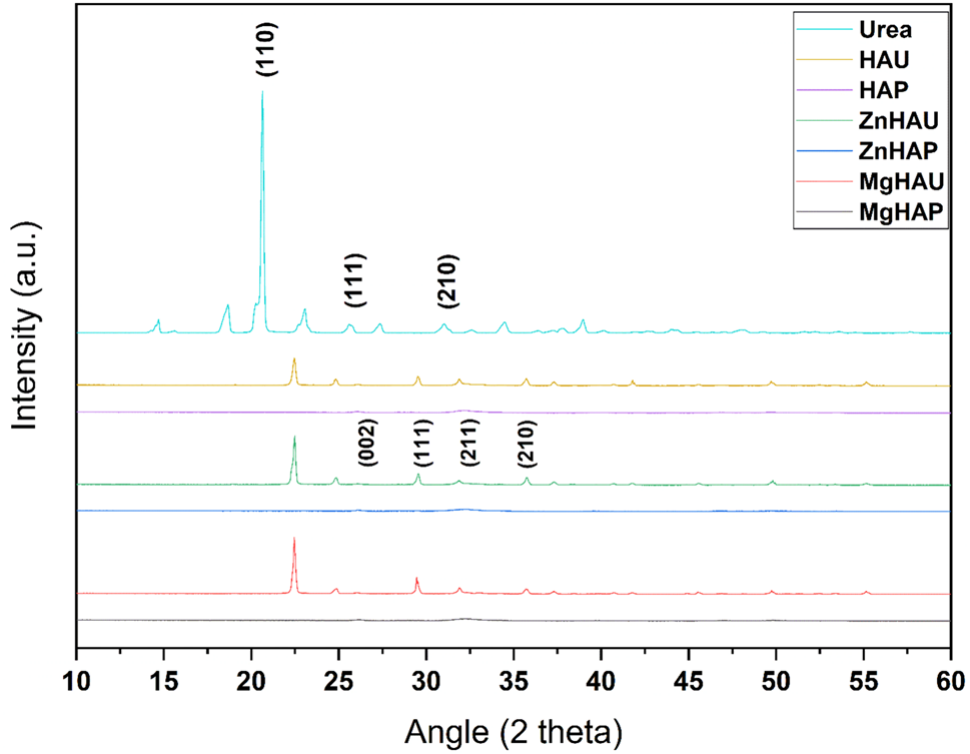
Powder X-ray diffraction (PXRD) analysis of the synthesized nanohybrids (HAU, MgHAU, and ZnHAU), bare nanoparticles (HAP, MgHAP, and ZnHAP), and urea.

XRD data revealed the polycrystalline nature of the undoped and magnesium, and zinc doped hydroxyapatite nanoparticles with the multiple peaks in the XRD patterns observed with maximum intensity at (211) miler indices of the crystal lattice (Fig. 1)^39,40^. The magnesium and zinc doping in the hydroxyapatite structure degraded the crystallinity and size of the nanostructures. The replacement of smaller radius atoms of magnesium ions (0.072 nm) and zinc ions (0.074 nm) with higher radius atom calcium ions (0.099 nm) in the hydroxyapatite structure at a few sites induced rearrangement of an intramolecular interaction in the backbone. Doping with zinc and magnesium generates distorted crystallinity in the hydroxyapatite, evidenced by smaller peak intensities and peak widening of (211 and 002) miler indices that reduced the size of the nanoparticles^41,42^. The smaller nanostructures exhibit a higher surface area that may be utilized to functionalize a large number of molecules. Therefore, the urea molecules have been functionalized on the surface of undoped and doped hydroxyapatite nanostructures. The XRD patterns of the urea nanohybrids showed urea characteristic peak intensities (210, 111, 110). The introduction of urea molecules on the surface of hydroxyapatite nanoparticles shifted the (210, 111) planes that caused structural modifications. The crystalline size of the HAP, MgHAP, and ZnHAP was 5.6 nm, 5.1 nm, and 4.95 nm, respectively. The crystalline size increased to 38.7 nm, 28.3 nm, and 20.8 nm for HAU, MgHAU, and ZnHAU, respectively.

**Figure 2:**
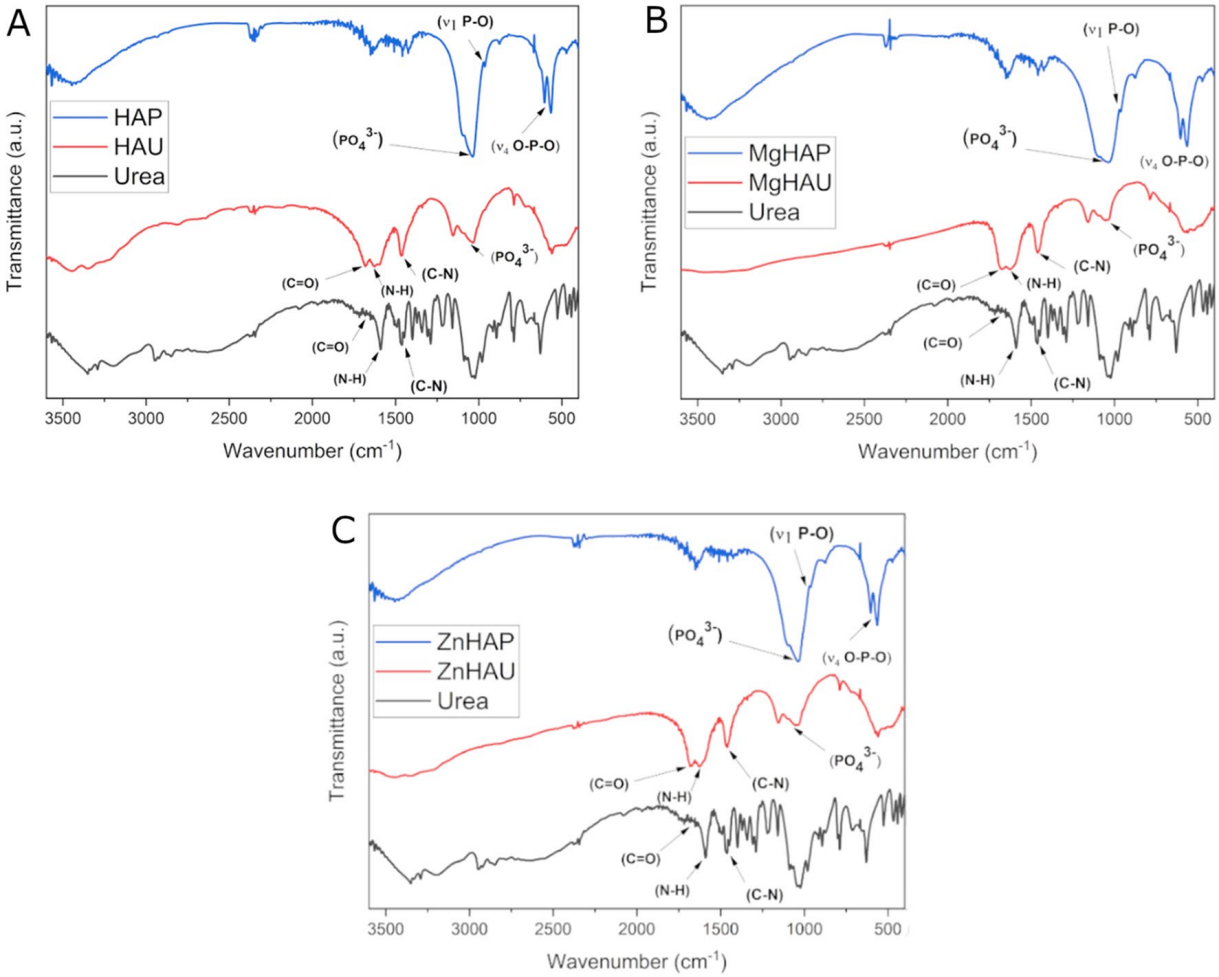
Fourier Transform Infrared (FTIR) spectrum of the synthesized nanohybrids. (**A**) HAP, HAU, and urea) (**B**) MgHAP, MgHAU, and urea (**C**) ZnHAP, ZnHAU, and urea. The FTIR spectrums show urea (C–N, N–H, C = O) and hydroxyapatite (PO_4_^3−^, P–O, O–P–O) distinct functional groups.

The FTIR (Fourier-transform infrared spectroscopy) analysis of the bare nanoparticles and urea nanohybrids revealed distinct functional groups of urea molecules (C–N, N–H, C=O) and hydroxyapatite nanoparticles (PO_4_^3−^, P–O, O–P–O) (Fig. 2A–C)^43,44^. The doping of the hydroxyapatite nanoparticles with zinc and magnesium induced a slight spectral shift of the O–P–O and PO_4_^3−^ functional groups compared to undoped hydroxyapatite nanoparticles (Fig. 2B,C). The calcium ion replacement with the zinc or magnesium caused rearrangements of intramolecular interactions in the doped nanoparticles and structural changes in the hydroxyapatite nanoparticles. The υ_1_ symmetric P–O modes stretching at 959–962 cm^−1^ and weak bonding O–H stretching region around 3300–3400 cm^−1^ were identified in all the nanoparticles. A spectral peak absorption and shifts in the C=O (1676–1682 cm^−1^), N–H (1623–1625 cm^−1^), and C–N (1461–1464 cm^−1^) functional groups in all three urea nanohybrids were observed (Fig. 2A–C)^38,43^.

The spectral shifts in the bands corresponding to C=O and C–N were noticed in the nanohybrids due to the weak chemical bonding environment and changes in the dipole movements. The spectral peak shift in N–H (3332–3353 cm^−1^) and peak widening were observed in nanohybrids compared with urea, indicating an induced alteration in the hydroxyapatite nanostructures. The phosphate sites of the hydroxyapatite nanohybrids were observed with reduced intensities compared with bare hydroxyapatite nanoparticles (Fig. 2A). The doping-inspired intramolecular interactions altered the υ_4_ vibrations and caused the spectral shifts of the phosphate bands. The chemical interaction with urea occupied the phosphate sites in the nanohybrids. The data indicated interaction between hydroxyapatite and urea, possibly through hydrogen bonding.

The hydroxyapatite nanostructures were observed with a rod-shaped and elongated crystalline with approximately 100–120 nm length and 25–35 nm width (Supplementary Fig. S1A). The nanoparticles exhibited a hydrophilic surface that frequently interacted with other hydroxyapatite nanoparticles to form an elongated spindle-like cluster^40,45,46^. The urea-hydroxyapatite nanohybrids were unorganized round-shaped nanostructures with an average diameter of approximately 125–140 nm (Supplementary Fig. S1B). The magnesium and zinc doped hydroxyapatite and urea nanohybrids exhibited a similar and irregular round-shaped structure with a diameter of approximately 70–100 nm than hydroxyapatite-urea (Supplementary Fig. S1C,D). The hydrophilic surface of doped and undoped hydroxyapatite nanoparticles attracts and accommodates urea molecules through weak interaction.

**Figure 3:**
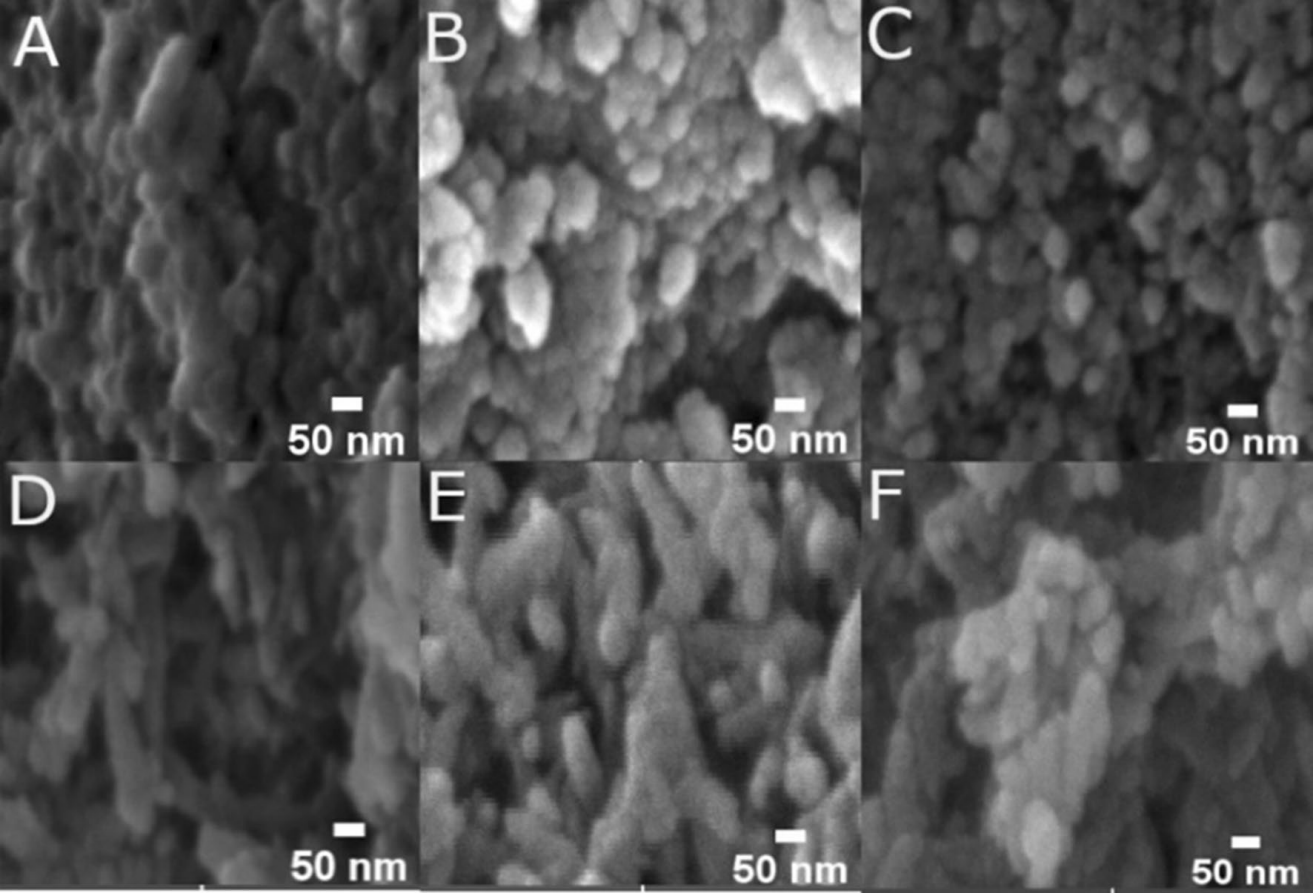
Scanning electron microscopy (SEM) images of the synthesized nanoparticles. (**A**) Hydroxyapatite (HAP), (**B**) Magnesium doped hydroxyapatite (MgHAP), (**C**) Zinc doped hydroxyapatite (ZnHAP), (**D**) Hydroxyapatite-urea (HAU), (**E**) Magnesium doped hydroxyapatite-urea (MgHAU), and (**F**) Zinc doped hydroxyapatite-urea (ZnHAU).

The scanning electron micrographs of the bare and urea-nanohybrids revealed unorganized irregular-shaped nanostructures (Fig. 3A–F). The continuous layers of irregular and spindle-like elongated structures were observed in the undoped and doped hydroxyapatite-urea nanohybrids. The doped and undoped hydroxyapatite nanoparticles appeared as smaller rod-shaped structures that were merged to form unorganized bead-like clusters^45,46^. The interaction with urea molecules on the surface of the bare hydroxyapatite nanoparticles changed the morphology and turned into larger elongated rod-shaped structures when coated with urea molecules. The diameter of the urea-nanohybrids (doped and undoped) was increased to approximately 100 nm compared to the 50–70 nm size of the bare nanoparticles. Energy-dispersive X-ray analysis of the nanohybrids confirmed the presence of calcium, phosphorus, magnesium, zinc, and nitrogen elements^38^.

The UV–visible absorption spectrum of the urea nanohybrids revealed an absorption peak shift to a shorter wavelength in doped hydroxyapatite-urea nanohybrids (Supplementary Fig. S2). The absorption peak shift could be signatures of electronic transitions introduced by the crystal reduction and bonding rearrangement in the urea-coated magnesium and zinc doped hydroxyapatite nanohybrids^47^. The free-electron oscillation of the zinc and magnesium could alter the energy levels of the urea-coated hydroxyapatite^48,49^.

The dynamic light scattering (DLS) analysis revealed the average particle size and zeta potential of bare and urea-coated hydroxyapatite nanoparticles (Supplementary Table S1). The undoped hydroxyapatite nanoparticles were 303.3 ± 54.2 nm, which was reduced for magnesium and zinc doped hydroxyapatite nanoparticles to 138.9 ± 17.8 nm and 206.5 ± 38.6 nm, respectively, and support the observations from XRD and TEM analysis. The average hydrodynamic diameter of the urea-coated hydroxyapatite nanohybrids was around 605.2 ± 83.7 nm, while magnesium and zinc doped hydroxyapatite-urea nanohybrids were found to be 325.1 ± 52.6 and 406.7 ± 41.4 nm, respectively. The zeta potential of the hydroxyapatite, magnesium, and zinc doped hydroxyapatite nanoparticles was in agreement with the previous report^50^ and was 18.9 mV, 22.6 mV, and 15.2 mV, respectively. The zeta potential of the nanoparticles was altered to − 4.6 mV, − 12.3 mV, and − 7.1 mV in the hydroxyapatite-urea, magnesium-doped hydroxyapatite-urea, and zinc-doped hydroxyapatite-urea, respectively after urea adsorption.

### Urea slowly released from nanohybrids in water

**Figure 4:**
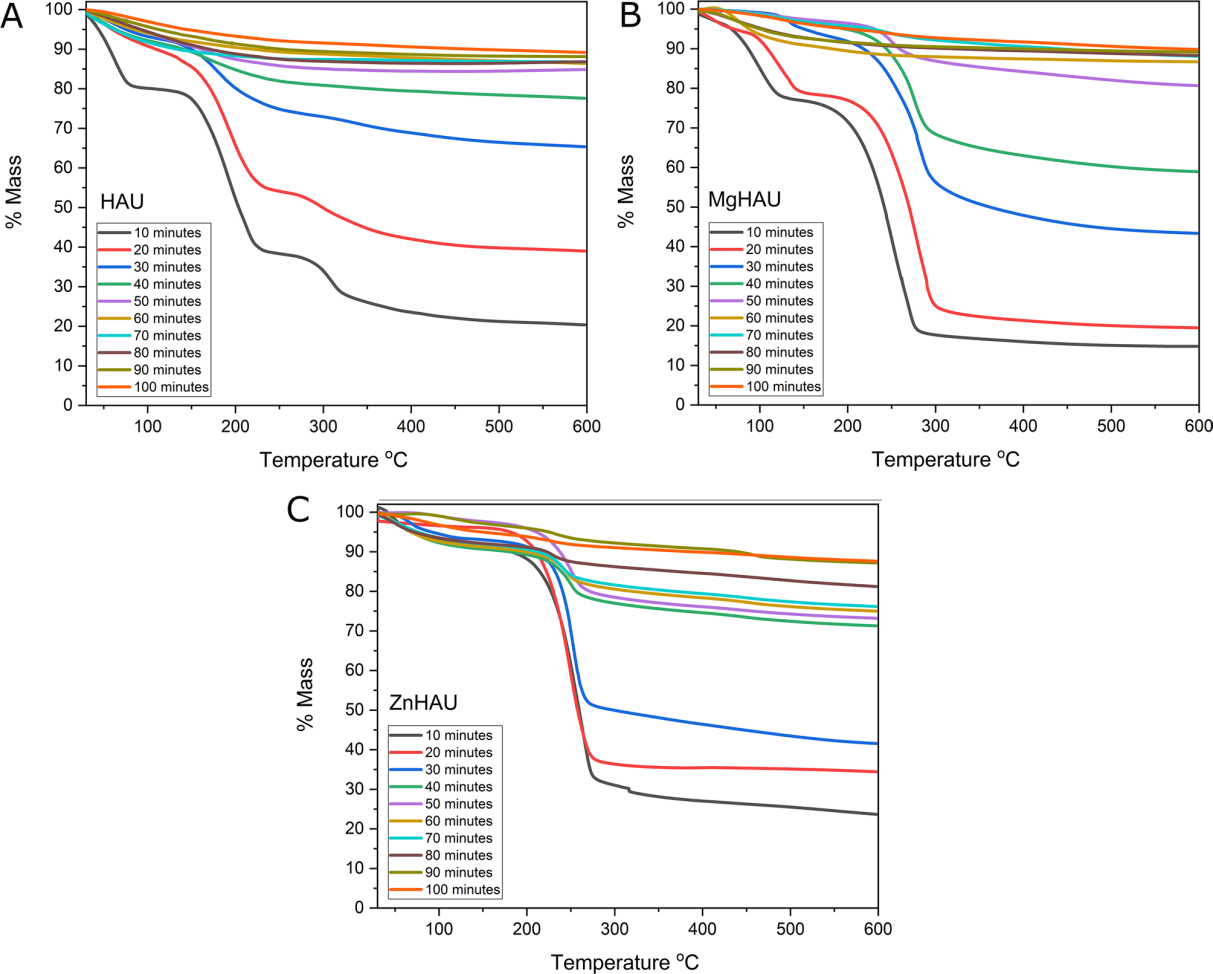
Thermogravimetric analysis of urea release from (**A**) HAU, (**B**) MgHAU, and (**C**) ZnHAU nanohybrids in a water environment (up to 100 min).

Urea, HAU, MgHAU, and ZnHAU were incubated directly with nuclease-free water and subjected to thermogravimetric analysis at 10 min intervals of up to 100 min. Hydroxyapatite is thermally stable and does not degrade up to 1200 ℃, while solid urea completely degrades around 350 ℃. TGA profiles of nanohybrids generated a release pattern of urea in the water (Fig. 4A–C). Urea was entirely released into the water within the first 2 min, and we did not find any pellet to perform TGA analysis. The HAU nanohybrid slowly released approximately 90% urea into the water within 50 min. Urea molecules were released faster in the water when a nanohybrid had a high concentration of urea molecules on its surface. Further, the amount of urea released reduced as urea molecule concentration on the nanohybrid diminished. The urea molecules were attached to the hydroxyapatite nanoparticles so firmly that the remaining 15% urea molecules were released after prolonged incubation of 100 min or more. The MgHAU showed a slow release of the urea for more than 100 min, and approximately 85% of urea was released within an hour of incubation with water.

The ZnHAU nanohybrid showed slow release of urea up to three initial incubations thereafter; it released a large amount, followed by the slow release of the urea up to 100 min. We observed that approximately 70% of urea was released within 50 min of incubation in water. The zinc and magnesium doping in the hydroxyapatite nanoparticles changed the urea release pattern in the water environment. In our previous study, we performed a release study in the soil matrix where the urea release from nanohybrids was observed for more than seven days^38^. Interestingly, the nanohybrids retained urea for longer than granular urea and facilitated prolonged release. Direct exposure of nanohybrids to water along with a vortex rapidly released urea from nanoparticles, but still, it is prolonged compared with urea granules in the same conditions where urea was entirely released into the water within 2 min. The results indicated that urea release from nanohybrids was determined by the surrounding concentration of water molecules and could be explained by density functional theory^38^.

The urea release was further slowed after 50 min of incubation, followed by a very slow release up to 100 min. FTIR data suggested that weak interactions such as hydrogen bonds could be a major player in the slow release of urea from nanohybrids, but the exact mechanism of slow release remains unknown. The bonding between urea and hydroxyapatite nanoparticles became stronger when fewer urea molecules were present on the nanoparticles. In our previous report, the prolonged urea release of up to one week from nanohybrids could result from such interactions^38^.

### Nanohybrids influence wheat leaf nitrogen uptake and plant growth

The synthesized nanohybrids were harvested through the following two methods; 1) the reaction mixture was directly dried at 65 ℃ after nanohybrids synthesis (Suspension dried) 2) the nanohybrids were harvested through centrifugation and dried at 65 ℃ after nanohybrids synthesis (Pellet dried). The nitrogen loading was higher in Suspension dried nanohybrids compared to Pellet dried nanohybrids. The nitrogen loading was 36.1%, 42.3%, and 41.2% in HAU, MgHAU, and ZnHAU suspension dried nanohybrids, and the Pellet dried HAU, MgHAU, and ZnHAU exhibited 22.4%, 26.3%, and 25.8%, respectively.

**Figure 5:**
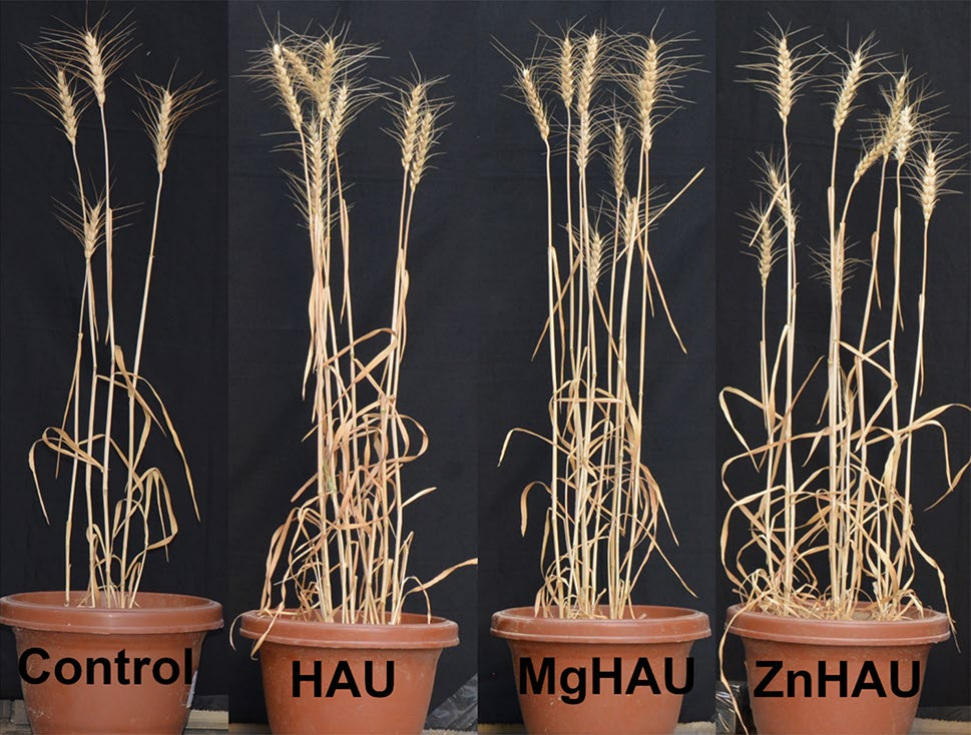
Wheat plants (at maturity) treated with nitrogen fertilizers, left to right: Control (Urea Fertilizer-100% RD_NPK_), HAU (Hydroxyapatite-Urea 50% Suspension-50% RD_N_ + 100% RD_PK_), MgHAU (Mg doped-Hydroxyapatite-Urea 50% Suspension-50% RD_N_ + 100% RD_PK_), and ZnHAU (Zn doped-Hydroxyapatite-Urea 50% Suspension-50% RD_N_ + 100% RD_PK_) in a pot experiment setup.

The agronomic traits of nanohybrids-treated wheat plants improved significantly compared to traditional urea-treated plants (Supplementary Fig. S3 and Fig. 5). Suspension nanohybrids treated wheat plants were observed with better agronomic trait characteristics than Pellet nanohybrids. No significant change in the plant height in nanohybrid treatments compared to control treatments was noticed except for MgHAU-50S, where plant height was significantly high (Supplementary Fig. S3A). The spike length was significantly increased up to 23.68% in the suspension nanohybrids and 21.92% in ZnHAU-25P treated wheat (Supplementary Fig. S3B). Likewise, spike weight was increased up to 78.95% in the MgHAU, and ZnHAU nanohybrids treated wheat, suggesting better growth and development of spikes (Supplementary Fig. S3C). Doped urea nanohybrids enhanced zinc and magnesium intake and boosted spike development^51^. The stem weight was improved (up to 105.8%) in the suspension doped urea nanohybrids treated wheat plants compared to control (Supplementary Fig. S3D).

Besides the nitrogen delivery, the zinc and magnesium doped hydroxyapatite may interfere with wheat plant metabolism to influence growth and development. The nanohybrids treatments enhanced the spikelet count to 85.37% compared to urea-treated wheat. The spikelet number was highest in the 50% nitrogen as suspension nanohybrids treated wheat, followed by 50% nitrogen as pelleted nanohybrids treatments (Supplementary Fig. S4A). A remarkable surge in the grain count, grain weight per pot, and 100-grain weight in the nanohybrid treatments was recorded. Grain count was significantly enhanced in suspension nanohybrids treated wheat (up to 121.56%) and the nanohybrid pellet treatments (up to 75.12%) (Supplementary Fig. S4B). The grain count increased in the half-nitrogen doses as nanohybrid treatments and MgHAU-S25, ZnHAU-S25, and ZnHAU-P25. The 100-grain weight was boosted as much as 2-fold in the nanohybrids treated wheat plants (Supplementary Fig. S4C).

The grain weight per pot of 25% nitrogen nanohybrids doses was higher than urea-treated plants. The maximum yield increment of 141.1% was noticed in the MgHAU-S50 treatments (Supplementary Fig. S4D). Previous studies noticed that slow-release urea had a beneficial impact on winter wheat crops^52,53^. ﻿The results agree with earlier reports that urea improved the wheat growth parameter when supplied as nanohybrids^38,54^﻿. The half and quarter doses of nitrogen as nanohybrids significantly hike the growth and yield characteristics of the wheat plants (Fig. 5). The synthesized nanohybrids can be used as an alternative nitrogen fertilizer that substantially cuts the nitrogen application to the agricultural fields^38^.

### Nanohybrids promote nutrient uptake in wheat

The nanohybrids used in the study are composed of calcium, phosphorus, nitrogen, magnesium, and zinc nutrients that may act as a multi-nutrient complex for plants. Since hydroxyapatite base is biodegradable and loosely organized, the doped and undoped nanohybrids can also release other compositional nutrients^38,55,56^. The nitrogen uptake was enhanced in wheat's grain, stem, and root tissues in the nanohybrids treatments (Table 1). Nitrogen uptake in the grain is a significant crop growth and quality parameter^57^. Grain nitrogen was boosted by 7.6–52.12% in all the nanohybrid treatments (Table 1).

**Table 1:** Macro-nutrients (N, P, K) composition in nanohybrids treated wheat plants.

Treatments	Wheat macro-nutrients (N, P, K)								
	Nitrogen (%)			Phosphorus (mg/kg)			Potassium (mg/kg)		
	Grain	Stem	Roots	Grain	Stem	Roots	Grain	Stem	Roots
No fertilizer	1.25 ± 0.06	0.53 ± 0.01	0.25 ± 0.005	775.43 ± 4.06	443.57 ± 4.73	430.88 ± 18.8	1571.1 ± 44.9	4187 ± 119.4	1067 ± 61.8
Fertilizer (100% RD_NPK_)	2.25 ± 0.11	0.63 ± 0.01	0.70 ± 0.02	1617.3 ± 10	808.8 ± 5.97	976.8 ± 6.6	3313.8 ± 51.4	7326.6 ± 42.8	2191 ± 95.4
Hydroxyapatite-urea 50% suspension (50% RD_N_ + 100% RD_PK_)	3.01 ± 0.26****	0.88 ± 0.18****	0.91 ± 0.009****	2024.2 ± 7.5****	426.4 ± 6.3****	630.75 ± 14.0****	2903.8 ± 45.6****	8100.5 ± 58.2****	3296 ± 75.5****
Magnesium doped-hydroxyapatite-urea 50% suspension (50% RD_N_ + 100% RD_PK_)	3.43 ± 0.29****	1.40 ± 0.05****	1.71 ± 0.01****	2931.6 ± 25.8****	492.2 ± 2.8****	985.6 ± 16.8 ns	2943.3 ± 75.3****	9272.2 ± 68.1****	2531 ± 57.1****
Zinc-Hydroxyapatite-urea 50% suspension (50% RD_N_ + 100% RD_PK_)	3.41 ± 0.33****	2.29 ± 0.18****	1.42 ± 0.05****	1977.8 ± 25.5****	1013.2 ± 19.3****	1179.9 ± 27.3****	3075.5 ± 40.5****	8828.3 ± 88.5****	2395 ± 57.9****
Hydroxyapatite-urea 25% suspension (25% RD_N_ + 100% RD_PK_)	2.51 ± 0.19 ns	1.53 ± 0.09****	0.35 ± 0.02****	1652.8 ± 36.5 ns	712 ± 10.1****	1049.4 ± 23.9****	2826.1 ± 58.4****	8643.3 ± 47.1****	2372 ± 60.7****
Magnesium doped-hydroxyapatite-urea 25% suspension (25% RD_N_ + 100% RD_PK_)	2.56 ± 0.21 ns	1.59 ± 0.07****	0.33 ± 0.004****	1934.2 ± 44.9****	689.5 ± 8****	936.4 ± 11.2****	3347.2 ± 34.3 ns	8002.7 ± 113****	2336 ± 58.2****
Zinc-hydroxyapatite-urea 25% suspension (25% RD_N_ + 100% RD_PK_)	2.8 ± 0.25***	2.2 ± 0.1****	0.8 ± 0.01****	1758.7 ± 33.9****	526.2 ± 10.6****	921.9 ± 17.4****	2866.1 ± 43.5****	7325.5 ± 94.3 ns	2246 ± 63.3 ns
Hydroxyapatite-urea 50% pellet (50% RD_N_ + 100% RD_PK_)	2.91 ± 0.28****	0.72 ± 0.05 ns	1.38 ± 0.02****	2776.5 ± 13.5****	1064.6 ± 13****	932.6 ± 20.8****	2672.2 ± 52.3****	8802.7 ± 85.8****	3915 ± 65.8****
Magnesium doped-hydroxyapatite-urea 50% pellet (50% RD_N_ + 100% RD_PK_)	3.27 ± 0.37****	0.7 ± 0.01 ns	2.52 ± 0.03****	2237 ± 42.4****	1252.7 ± 14.4****	714.6 ± 17.1****	2551.1 ± 39.5****	7663.3 ± 56.4****	2233 ± 58.3 ns
Zinc-hydroxyapatite-urea 50% pellet (50% RD_N_ + 100% RD_PK_)	3.11 ± 0.33****	0.7 ± 0.009 ns	2.83 ± 0.06****	1870.4 ± 26.8****	956.7 ± 23.1****	1743.9 ± 17.3****	2966.6 ± 39.3****	7191.1 ± 63.1**	2710 ± 43.7****
Hydroxyapatite-urea 25% pellet (25% RD_N_ + 100% RD_PK_)	2.34 ± 0.28 ns	0.67 ± 0.02 ns	1.45 ± 0.09****	1616.2 ± 29.6 ns	840.6 ± 21.5****	1046.1 ± 18.8****	2711.1 ± 54.6****	7475.5 ± 34.8***	2325 ± 10***
Magnesium doped-hydroxyapatite-urea 25% pellet (25% RD_N_ + 100% RD_PK_)	2.42 ± 0.31 ns	0.70 ± 0.07 ns	0.63 ± 0.01**	1683.5 ± 31.8****	744.9 ± 15.3****	1432.2 ± 24.5****	3252.2 ± 53.2 ns	7198.8 ± 51.7**	2151 ± 85.8 ns
Zinc-hydroxyapatite-urea 25% pellet (25% RD_N_ + 100% RD_PK_)	2.68 ± 0.37*	0.65 ± 0.02 ns	1.67 ± 0.04****	1676.6 ± 36.1***	919.3 ± 8.9****	750.4 ± 14****	2985 ± 77.6****	7030 ± 97.2****	2534.4 ± 31.6****

The levels of nutritional elements (nitrogen, phosphorus, potassium) in the wheat stem, and root tissues after harvesting of the wheat crop treated with a quarter and half dose of nitrogen as nanohybrids (suspension and pellet), full dose of nitrogen as urea (as control), and without fertilizer (No Fertilizer). The values are provided as mean ± standard deviation, and statistical significance was calculated by one-way ANOVA with Dunnett’s multiple comparison test. The letters ‘*’, ‘**’, ‘***’, and ‘****’ represent ‘p < 0.05’, ‘p < 0.01’, ‘p < 0.001’, and ‘p < 0.0001’, respectively and ‘ns’ represents ‘not significant’.

Interestingly, both forms of 50% nitrogen doses, suspension and pellet, assisted in higher nitrogen uptake. A significantly higher uptake was seen in the stem part of wheat plants treated with a suspension of nanohybrids, while pelleted nanohybrids maintained the same nitrogen content as control (Table 1). ZnHAU-50 and MgHAU-50 doses spiked the nitrogen content up to three times in the root tissues. Nitrogen accumulation in the wheat suggested timely nitrogen availability throughout the wheat life cycle^57–59^. Grain nitrogen was enhanced irrespective of suspension or pellet form used as nitrogen fertilizer. A half and quarter nitrogen doses as nanohybrids effectively fulfilled the wheat plant's nitrogen requirement.

Nitrogen fertilization impacts the grain phosphorus uptake in wheat plants^60^. Grain phosphorus content was dramatically increased exclusively in all the MgHAU treatments. The phosphorus content was increased (up to 81.26%) in the nanohybrids treated wheat grains compared with control plants (Table 1). The pellet HAU treatments showed better phosphorus uptake in the stem tissues, while ZnHAU-P50 and MgHAU-P25 showed substantial phosphorus accumulation in root tissues (Table 1). The increased phosphorus levels indicated either degradation of the nanomaterials releasing phosphorus or the uptake of nanoparticles. The nanohybrid may also act as a good source of phosphorus fertilizer^61^. The potassium content was significantly improved in the stem (up to 26.55%) and root tissues treated with the nanohybrids, but the grain potassium content was slightly reduced in all the nanohybrid treatments (Table 1). The HAU-50 treatments promoted potassium accumulation in wheat root tissues. The calcium uptake was increased by approximately 101.39% in the suspension ZnHAU-50 treated grains. The suspension nanohybrids encouraged calcium uptake in wheat grain tissues (Supplementary Table S2), but the calcium levels declined to 34.43% in the nanohybrid-treated stem tissues. The calcium content was maintained in the 50% nitrogen as nanohybrids doses while substantially reducing 25% nitrogen as nanohybrids doses compared to control plants (Supplementary Table S2). The magnesium content was improved significantly in most of the nanohybrids treated grains with the maximum surge in the MgHAU-50 treatments (up to 103%) (Supplementary Table S2). Except for ZnHAU-50S, all the nanohybrids treated wheat stems were found to have lower magnesium levels than control.

Zinc accumulation was raised in all the nanohybrids treatments, but a significant improvement of up to 85.81% in the pellet nanohybrids treated grains compared with control was recorded (Supplementary Table S3). The zinc content was decreased in the nanohybrid-treated stem (up to 57.27%) and root tissues (up to 66.19%) compared with the control. The zinc was efficiently absorbed and delivered to the grain tissues. The distribution of the nutrient elements in winter wheat determines the yield parameters and is affected by nitrogen fertilization management^62^. The redistribution and accumulation of the nutrient elements are vital for wheat plant growth. The iron content of the nanohybrid treated grains was notably upraised up to 89.93% in the nanohybrid treatments. The grain iron content was enhanced in 50% nitrogen doses as nanohybrids treatments, and the maximum accumulation was observed in the pellet nanohybrids treated grains (Supplementary Table S3).

Iron content was increased exclusively in the stem tissues treated with HAU-S50, HAU-S25, MgHAU-P25, and ZnHAU-P25 nanohybrids, while root tissues treated with MgHAU-S50, HAU-S25, MgHAU-P50, and ZnHAU-P50 showed better iron accumulation. The manganese content was exceptionally built up in the 50% nitrogen nanohybrid treated grain tissues (Supplementary Table S3) up to 85.6% increment compared to urea treatments. Manganese level in the stem tissues was reduced in all the nanohybrid treatments. Compared to control, the manganese content in the root tissues was elevated in MgHAU-S50, HAU-S25, MgHAU-P50, and ZnHAU-P50 treatments.

**Figure 6:**
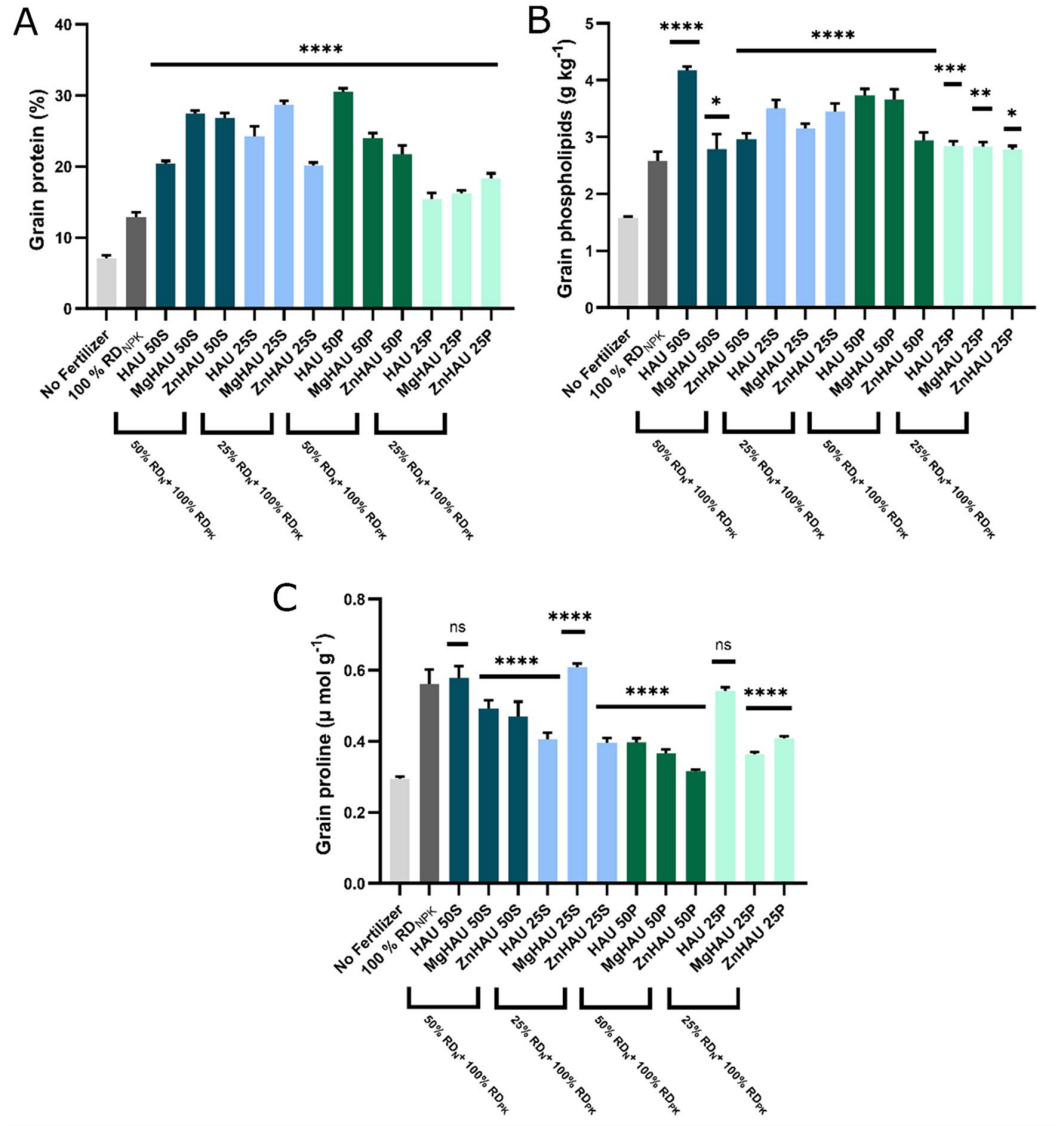
Wheat grain quality parameters evaluated after harvesting. (**A**) Protein, (**B**) Phospholipids, and (**C**) Proline were evaluated for the wheat grains after harvesting in the wheat crop treated with a quarter and half dose of nitrogen as nanohybrids (suspension and pellet), full dose of nitrogen as urea (as control), and without fertilizer (no fertilizer). All the nanohybrid treatments (HAU: Hydroxyapatite-urea, MgHAU: Mg-doped hydroxyapatite-urea, and ZnHAU: Zn-doped hydroxyapatite-urea) were compared with the control or 100% RD_NPK_ treatment. The values are provided as mean ± standard deviation, and statistical significance was calculated by one-way ANOVA with Dunnett’s multiple comparison test. The letters ‘*’, ‘**’, ‘***’, and ‘****’ represent ‘p < 0.05’, ‘p < 0.01’, ‘p < 0.001’, and ‘p < 0.0001’, respectively and ‘ns’ represents ‘not significant’.

Here, iron and manganese were not supplied as fertilizer to the crop, but still, an accumulation was observed, suggesting biosorption through hydroxyapatite matrix^63,64^. Due to their larger surface area, the hydroxyapatite nanomaterials could adsorb metal ions such as iron and manganese from the soil that was eventually taken up by the wheat plant^65^. This phenomenon revealed the additional benefits of using a hydroxyapatite matrix for delivering nutrient molecules. A significant improvement was observed in the total protein content of the nanohybrids treated plants compared to no fertilizer and only (NPK) fertilizer treatments. The protein content was increased 2-fold in all the nanohybrid treatments except for 25% pellet nanohybrid, where the increment was noticed up to 50% (Fig. 6A). The slow release of nitrogen positively impacts protein levels in wheat grains and grain quality^66^. Total phospholipid accumulated in the wheat grains is an essential measure of assessing grain quality^67,68^. The total phospholipid concentration increased significantly in most nanohybrid treatments (Fig. 6B). The maximum increment was observed in HAU-S50, HAU-S25, HAU-P50, and MgHAU-P50 by 51%, 22%, 38%, and 28%, respectively. Nanohybrid treatments promoted grain nutrition enrichment.

Interestingly, proline concentration was increased in HAU-S50, MgHAU-S25, and HAU-P25 by 12%, 20%, and 6.5%, respectively (Fig. 6C). All remaining treatments showed a decreased proline level compared to urea fertilizer-treated wheat grains. The proline concentration increased in response to stress conditions^69,70^.

**Figure 7:**
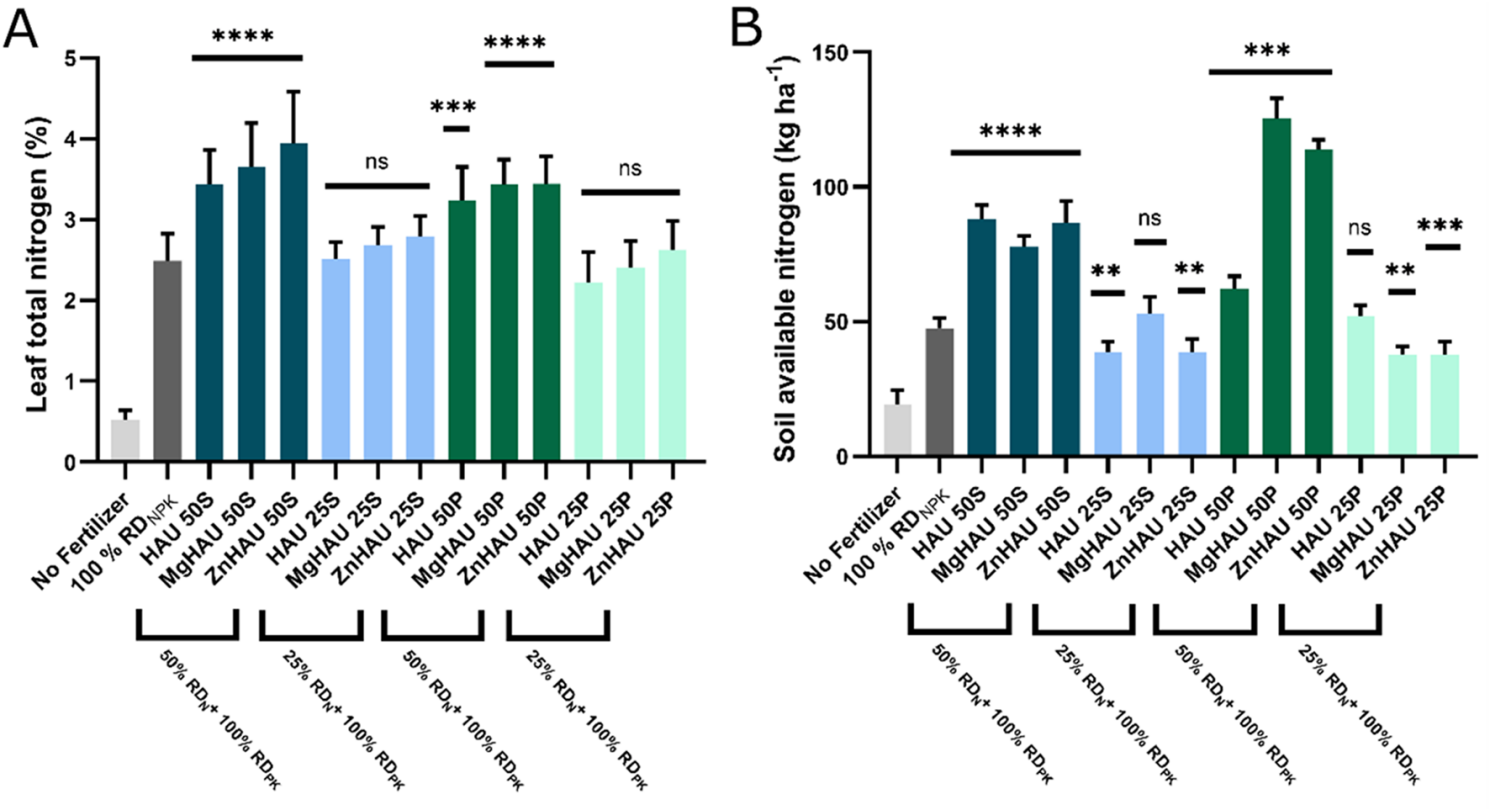
Wheat leaf nitrogen, and soil available nitrogen estimation. (**A**) Leaf nitrogen after 40 days of germination, and (**B**) Soil available nitrogen after the wheat crop cycle were evaluated in the wheat crop and soil treated with a quarter and half dose of nitrogen as nanohybrids (suspension and pellet), full dose of nitrogen as urea (as control), and without fertilizer (No Fertilizer). All the nanohybrid treatments (HAU: Hydroxyapatite-urea, MgHAU: Mg-doped hydroxyapatite-urea, and ZnHAU: Zn-doped hydroxyapatite-urea) were compared with the control or 100% RD_NPK_ treatment. The values are provided as mean ± standard deviation, and statistical significance was calculated by one-way ANOVA with Dunnett’s multiple comparison test. The letters ‘*’, ‘**’, ‘***’, and ‘****’ represent ‘p < 0.05’, ‘p < 0.01’, ‘p < 0.001’, and ‘p < 0.0001’, respectively and ‘ns’ represents ‘not significant’.

Wheat initial nitrogen uptake was assessed through nitrogen estimation of wheat leaf harvested at 40 days post-germination (Fig. 7A). The leaf nitrogen content analysis indicated nitrogen availability for plant uptake from soil^71,72^. Leaf nitrogen content was significantly enhanced (up to 58.35%) in all 50% nitrogen as the nanohybrids treated wheat plants compared with control (100% nitrogen as urea). 25% nitrogen as nanohybrids maintained the leaf nitrogen as control, suggesting that nanohybrids ensured timely availability of the nitrogen nutrient in the soil.

Plants require a small amount of total applied nitrogen; therefore, 25% nitrogen doses as suspension nanohybrids sufficiently deliver nitrogen at the early growth stage. The ZnHAU-50 treatments were found to be delivering the highest nitrogen among all three urea nanohybrids variants. It can be inferred that the interaction between urea and all three nanohybrids introduced sustainable release for a longer time^38^. The urea granules burst and release nitrogen mainly lost through volatilization and leaching over time^73,74^. The nanohybrids avoided the volatilization of nitrogen that can readily be available for plant uptake^75^.

The total available nitrogen was significantly higher in the half doses of suspension and pellet nanohybrids treated soil than in urea and other treatments (Fig. 7B). The available nitrogen levels were either maintained as in control treatments or reduced to some extent in 25% nitrogen doses of suspension and pellet nanohybrids. The results suggest the sustainable release of nitrogen from nanohybrids. A substantial amount of nitrogen was saved in the soil that could be used for consecutive crop cycles, thus improving the crop nitrogen utilization efficiency^38,76,54^.

The nanohybrids held nitrogen in the soil throughout the wheat life cycle and avoided possible nitrogen losses through volatilization and leaching^77^. The pot experiment revealed extraordinary improvements in wheat plant growth, yield, nutritional elements uptake, and grain quality in all three nitrogenous nanohybrid treatments. The half and quarter nitrogen as nanohybrid were sufficient to obtain plant growth, and nitrogen bioavailability was equivalent to 100% nitrogen as conventional urea fertilizers.

## Discussion

Nanomaterials are emerging as an alternative to bulk materials in agricultural applications due to their extraordinary physical and chemical properties. Nanoscale particles are being explored to resolve significant challenges associated with global food demand, environmental implications, and sustainable agricultural practices^78^. For instance, doped hydroxyapatite and urea^38^, zinc oxide^79^, iron and magnesium^80^, urea-hydroxyapatite^81^, potassium and phosphorus^82^, zinc and copper, and chitosan-PMAA-NPK^83^ nano-fertilizers have been developed for improving crop growth and yield. However, we still need comprehensive studies on developing effective nano-fertilizers, especially nitrogen nano-fertilizers, and assessing their impact on plant growth^30^. In addition, the biocompatibility of the nanoparticles must be ensured before their release into the environment, which is the crucial factor for adopting nanomaterials for agricultural and biological applications^20,84^.

In the present study, we focused on developing a nanocarrier system based on hydroxyapatite nanoparticles that delivered nitrogen to the plants while incorporating multiple plant nutrients as building blocks. Hydroxyapatite nanoparticles were chemically synthesized, doped with zinc and magnesium ions, and used as delivery vehicles for nitrogen nutrients to plants. Doping with Zn and Mg enhanced the properties of hydroxyapatite nanoparticles and served as additional nutrient supply to plants.

The XRD data analysis of the bare and urea-nanohybrids suggested that doping with zinc and magnesium introduced deformities in the hydroxyapatite structure that reduced the particle size. The smaller size of the doped nanoparticles was validated with TEM and SEM analysis compared to undoped hydroxyapatite. Smaller particles exhibit a higher surface area and accommodate more urea molecules on their surfaces. As reported earlier, FTIR analysis of nanoparticles confirmed the shift in the vibrational modes of C=O and C–N, N–H bonding indicating a weak interaction between distinct functional groups of urea and hydroxyapatite^85^. Urea molecules could bind on the hydroxyapatite surface through hydrogen bonds^81,85^. We demonstrated doping-induced changes in the hydroxyapatite structure that altered urea binding and release. The findings support the previous reports and indicate a weaker bonding between urea and hydroxyapatite nanoparticles. The hydrodynamic particle size of the doped and undoped hydroxyapatite nanoparticles was bigger than the one identified with microscopy because of the hydrophilic interactions^38^.

The wheat crop growth parameters and nutrient uptake showed significant improvement, notably in the 50% nitrogen doses as nanohybrids, and maintained the growth as control treatments in 25% nitrogen doses as nanohybrids. The previous studies showed that half dose of nitrogen as nanohybrids maintained the crop growth and nitrogen uptake as full nitrogen dose of control treatments^38^. In the present study, the 25% nitrogen dose as nanohybrids, both suspension, and pellet forms, maintained the nitrogen levels in the wheat grains as a control. It is possible only when nitrogen from nanohybrids is substantially and slowly available to plants for uptake. Doped hydroxyapatite nanoparticles have a large surface area that allows the adsorption of metal ions such as iron and manganese from the surrounding soil matrix. It is noteworthy that hydroxyapatite nanoparticles have been used as metal adsorbents to minimize heavy metal toxicity in wastewater^86,87^. Therefore, the doped and undoped hydroxyapatite-urea nanohybrids assisted with iron and manganese uptake and served as a multi-nutrient source to plants.

After the wheat crop life cycle, the soil available nitrogen levels were significantly high in all pellet nanohybrids and 50% suspension doses. Unlike suspension nanohybrids, the pellet nanohybrids lost urea molecules during the harvesting process and exhibited a very slow nitrogen release^38^. Therefore, suspension and pellet nanohybrids were assessed for optimum nitrogen release and plant availability. The leaf nitrogen uptake and grain protein content were improved in half nitrogen doses as nanohybrid treatments compared with control. Previous studies reported similar results and confirmed prolonged nitrogen availability in the soil due to the slower release of nitrogen from nanohybrids^38^. The present study provides detailed experimental evidence for the positive impact of hydroxyapatite-urea and its doped variants on the growth and productivity of wheat plants. This study demonstrated the potential of the hydroxyapatite nanoparticles as a vehicle for nutrient delivery and as a source of multiple nutrients.

## Conclusion

Doping of hydroxyapatite with magnesium and zinc induced structural changes and generated a multi-nutrient complex at the nanoscale to enhance the urea loading and influence the urea release. Doped hydroxyapatite nanoparticles can be a vital biocompatible system for delivering several macro- and micro-nutrients to plants. The half and quarter doses of urea nanohybrids enhanced the yield parameters, nutrient levels, and leaf nitrogen, reduced nitrogen loss, and ensured sufficient nitrogen availability during the early wheat growth stage. The urea coated on nanoparticles did not leach or volatilize from the soil and slowly delivered the nitrogen to wheat plants. As a result, the half and quarter dose of nanohybrids maintained wheat growth without provoking any negative impact. Further, a better understanding of the interactions between Zn and Mg-doped hydroxyapatite nanoparticles and urea molecules is needed to efficiently use this nano-formulation in agriculture. The impact of such nano-fertilizers on different crop models and soil conditions will be a vital parameter to consider before their widespread use in agriculture.

## Materials and methods

### Nanomaterial synthesis

The hydroxyapatite nanoparticles were synthesized using our previously reported method with a few modifications^38^. One molar calcium hydroxide aqueous solution was stirred at 400 rpm for half an hour to prepare the nanomaterial while maintaining the solution temperature of 50 °C. Further, 0.6 molars of phosphoric acid were added to the suspension dropwise, the mixture was stirred for 2 h, and the resulting suspension was dried at 65 °C for 10 h. Magnesium chloride (0.05 molar) and zinc sulfate (0.05 molar) were mixed with 0.95 molar calcium hydroxide separately, and 0.6 molars phosphoric acid was slowly added to the suspension to synthesize magnesium and zinc doped hydroxyapatite nanoparticles. The resulting suspension was stirred for 2 h at 50 °C and dried at 65 °C for 10 h.

Urea-coated hydroxyapatite nanoparticles were prepared by slowly dissolving seven molar urea in water and mixing with one molar calcium hydroxide with continuous stirring at 400 rpm for an hour, and the reaction temperature was adjusted to 50 °C. Further, 0.6 molars of phosphoric acid were slowly added to the suspensions. The resulting suspension was stirred for 3 h at 50 °C and dried at 65 °C for 24 h. For synthesising magnesium doped hydroxyapatite nanohybrids of urea, seven molar urea, 0.95 molar calcium hydroxide, and 0.05 molar magnesium chloride were mixed at 400 rpm for an hour, and the reaction temperature was adjusted to 50 °C. To synthesise zinc doped hydroxyapatite-urea nanohybrids, seven molar urea, 0.95 molar calcium hydroxide, and 0.05 molar zinc sulfate were mixed at 400 rpm for an hour, and the reaction temperature was adjusted to 50 °C. Later, 0.6 molars of phosphoric acid were slowly added to the suspensions, and the resulting suspension was stirred for 3 h at 50 °C. The suspensions of all three urea nanohybrids (hydroxyapatite-urea, magnesium doped hydroxyapatite-urea, and zinc doped hydroxyapatite-urea) were collected through two schemes. In the first scheme, all the nanohybrid suspensions were directly dried at 65 °C for 24–36 h in a hot air oven. The second scheme included centrifugation of the nanohybrid suspensions at 10,000 rpm for 30 min at room temperature. The supernatant was discarded, and the pellet was dried in a hot air oven at 65 °C for 24–36 h. Finally, the dried powder was used for nitrogen estimation and fertilizer application.

### Nanomaterial characterization

#### Powder X-ray diffraction (PXRD)

Powder X-ray diffraction (PXRD) of the dried samples was carried out on a Bruker D8 Advance diffractometer. The monochromatic Ni-filtered Cu Kα radiation (λ = 1.54 Å) was used for the analysis.

#### Fourier transform Infrared (FTIR)

FTIR analysis was performed on a Varian 7000 FTIR machine, and the ATR-FTIR spectrum was obtained in the region of 4000–400 cm^−1^.

#### Cryo-transmission electron microscopy (Cryo-TEM)

TEM images of the samples were obtained on a TALOS cryo transmission electron microscope at an accelerating voltage of 200 kV. The samples were dispersed in MiliQ water, sonicated for 15 min, drop cast on carbon-coated copper grids (300 mesh), and incubated for 1 h at room temperature.

#### Scanning electron microscopy (SEM)

The SEM images of the dried samples were acquired on the TESCAN LYRA3 machine, a focused ion beam scanning electron microscope. The dried sample powder was sprinkled on a black carbon tape surface on steel grids, and the samples were coated with ultrathin electrically conducting gold metal before imaging.

#### UV–visible spectrophotometry (UV–Vis)

UV–Vis spectra of the synthesized bare particles and nanohybrids were obtained on a Labman UV–visible spectrophotometer.

#### Hydrodynamic particle size and zeta potential

The average hydrodynamic diameter and zeta potential of the synthesized urea-nanohybrids and bare nanoparticles using the Horiba Particle Analyzer SZ-100V2 instrument.

### Nitrogen release in the water

1 gram oven-dried nanohybrids or urea were incubated and subjected to vortex with nuclease-free water at 25 °C (pH at 6.9), and the resulting suspension was centrifuged for 5 min at 12,000 rpm^88^. The pellets were oven-dried for 24 h at 60 °C and subjected to thermogravimetric analysis to indirectly estimate urea release through mass loss. The synthesized HAU, Mg-HAU, and Zn-HAU were incubated with water at 10 min times intervals of up to 100 min. The difference between the residual mass and effective initial mass was considered for the total urea loss from nanohybrids calculated by the following equation:

$$\% Total\,urea\,loss = \frac{Effective\,initial\,mass - Residual\,mass}{{Initial\,mass}} \times 100$$

where residual mass = weight of sample at the end of the TGA cycle, initial mass = total sample weight subjected to TGA cycle, moisture mass = weight of water molecules (mass loss up to 120 °C), effective initial mass = initial mass − moisture mass.

### Experimental soil

The experimental soil for the pot study was classified as Ustochrept in the order of Inceptisol. The surface experimental soil (0–15 cm) texture was a sandy clay-loam soil (50.8% sand, 23.2% silt, and 25.9% clay) determined by the hydrometer method^89^. It consists of 217 kg ha^−1^ alkaline permanganate oxidizable nitrogen (N)^90^, 12.0 kg ha^−1^ available phosphorus (P)^91^, 213 kg ha^−1^ 1 N ammonium acetate exchangeable potassium (K)^92^, and 5.3 g kg^−1^ organic carbon (C)^93^. The pH of the soil was 7.8 (1:2.5 soil: water ratio)^94^, and diethylene triamine penta acetic acid (DTPA)-extractable zinc (Zn)^95^ in soil was 0.61 mg kg^−1^ soil. Fifty kilograms of virgin surface soil from the experimental field were collected, passed through a 2 mm sieve, and used for the pot experiment.

### Plant growth and nitrogen treatments

The cultivation of plants and collection of the plant materials were performed per the guidelines provided by the Indian Agricultural Research Institute, India^38^. The wheat seeds (Pusa HD 3086) were surface sterilized with ultraviolet irradiation, incubated with 4% sodium hypochlorite for 10 min, and repeatedly washed with de-ionized water. A total of four seeds were placed at the 7–10 cm depth in the soil-filled plastic pot (20 cm height, 25 cm top diameter, and 19 cm base diameter). For wheat, a nitrogen dose of 150 kg N per hectare was used. The phosphorus and potassium were applied as single super phosphate (SSP-16% P_2_O_5_) (60 kg P_2_O_5_ per hectare) and muriate of potash (MOP-60% K_2_O) (60 kg per hectare), respectively, in all the treatments uniformly. The full dose of the phosphorus and potassium fertilizers was applied at the time of seed planting. Nitrogen fertilizers were applied in two instalments, half at the time of seed planting and the remaining half after four weeks of seed germination.

The nitrogen was applied as granular urea and nanohybrids. Our previous study demonstrated that 100% and 75% nitrogen as nanohybrids improved wheat crop growth, nitrogen uptake, and soil available nitrogen compared with 100% nitrogen as urea (control) treatments^38^. We observed a higher level of soil available nitrogen after the wheat crop cycle. Therefore, this work assessed the 50% and 25% nitrogen doses as nanohybrids against full-dose nitrogen as conventional urea. The nanohybrids (HAU-hydroxyapatite-urea, MgHAU-magnesium doped hydroxyapatite-urea, and ZnHAU-zinc doped hydroxyapatite-urea) were synthesized in two schemes, suspension (S) and pellet (P). Two dosages of each scheme treatment, 50% nitrogen (75 kg per hectare) and 25% nitrogen (37.5 kg per hectare) of the recommendation (150 kg nitrogen per hectare), were applied (Table 2).

**Table 2:** The fertilizers treatment scheme used in the study.

Treatments	
(i) No fertilizers	
(ii) Granular urea 100% RD_NPK_ (100% N dose)	
**Suspension nanohybrids**	
(iii) Hydroxyapatite-urea suspension 50% RD_N_ + 100% RD_PK_ (HAU 50S)	(vi) Hydroxyapatite-urea suspension 25% RD_N_ + 100% RD_PK_ (HAU 25S)
(iv) Mg-doped Hydroxyapatite-urea suspension 50% RD_N_ + 100% RD_PK_ (MgHAU 50S)	(vii) Mg-doped Hydroxyapatite-urea suspension 25% RD_N_ + 100% RD_PK_ ZnHAU 25S)
(v) Zn doped Hydroxyapatite-urea suspension 50% RD_N_ + 100% RD_PK_ (ZnHAU 50S)	(viii) Zn doped Hydroxyapatite-urea suspension 25% RD_N_ + 100% RD_PK_ (ZnHAU 25S)
**Pellet nanohybrids**	
(ix) Hydroxyapatite-urea Pellet 50% RD_N_ + 100% RD_PK_ (HAU 50P)	(xii) Hydroxyapatite-urea Pellet 25% RD_N_ + 100% RD_PK_ (HAU 50P)
(x) Mg-doped Hydroxyapatite-urea Pellet 50% RD_N_ + 100% RD_PK_ (MgHAU 50P)	(x) Mg-doped Hydroxyapatite-urea Pellet 25% RD_N_ + 100% RD_PK_ (HAU 50P)
(xi) Zn doped Hydroxyapatite-urea Pellet 50% RD_N_ + 100% RD_PK_ (ZnHAU 50P)	(xi) Zn doped Hydroxyapatite-urea Pellet 25% RD_N_ + 100% RD_PK_ (HAU 50P)

All the treatments were replicated three times.

The agronomic parameters: plant height, spike length, spike weight, stem weight, number of spikelets per spike, number of grains per spike, total kernel weight per pot, and 100-grain weight, were recorded after harvesting the crop. After harvesting, the wheat grain, straw, and root samples were dried at 70 °C and processed for elemental (phosphorus, nitrogen, potassium, calcium, magnesium, zinc, iron, manganese) and grain quality. Wheat leaves were collected after 40 days of germination and used for the nitrogen analysis.

### Elemental analysis

Total nitrogen estimation in the plant tissues was performed using the Kjeldahl method^96^. The total available nitrogen in the soil was estimated through the Kjeldahl method using alkaline potassium permanganate as an oxidative agent^90,97^. Total plant phosphorus was assessed using the phosphovanado-molybdate method^98^, total calcium^99^ and potassium^100^ estimation used a flame photometer, and magnesium estimation using the EDTA titration method^101^. Zinc, manganese, copper, and iron were estimated using an atomic absorption spectrophotometer^102,103^. Total grain protein^104^, phospholipid^105^, and proline^106^ content were determined to assess grain quality.

### Statistical analysis

Numerical data are provided as mean ± standard deviation, and statistical significance was calculated by one-way ANOVA with Dunnett’s multiple comparison test. The symbols ‘*’, ‘**’, ‘***’, and ‘****’ represent ‘p < 0.05’, ‘p < 0.01’, ‘p < 0.001’, and ‘p < 0.0001’, respectively and ‘ns’ represents data that is ‘not significant’.

## Supplementary Information

Supplementary Information.

## Data Availability

All data generated during the study has been provided in this manuscript and its supplementary data file.
