# Supplementary material for: Zinc- and magnesium-doped hydroxyapatite-urea nanohybrids enhance wheat growth and nitrogen uptake
Source: Sci Rep. 2022 Nov 14;12:19506. doi: 10.1038/s41598-022-20772-w (PMC9663570; doi:10.1038/s41598-022-20772-w)
Supplement: Supplementary file 1 — Supplementary Information. [file 41598_2022_20772_MOESM1_ESM.docx]

Zinc- and magnesium-doped hydroxyapatite-urea nanohybrids enhance wheat growth and nitrogen uptake

Bhaskar Sharma^ab^, Luis O. B. Afonso^a^, Manoj Pratap Singh^d^, Udit Soni^c^, David M. Cahill^a^

^a^ School of Life and Environmental Sciences, Deakin University, Geelong Waurn Ponds Campus, Geelong, VIC 3216, Australia

^b^ Department of Botany and Plant Sciences, University of California-Riverside, Riverside, California 92521, United States

^c^ Department of Biotechnology, TERI School of Advanced Studies, New Delhi, India 110070

^d^ AIRF, Jawaharlal Nehru University, New Delhi, India

**Correspondence:**

David M. Cahill − School of Life and Environmental Sciences, Deakin University, Geelong Waurn Ponds Campus, Geelong, VIC 3216, Australia; ORCID: <https://orcid.org/0000-0002-2556-0528>; Email: david.cahill@deakin.edu.au

Udit Soni – Department of Biotechnology, TERI School of Advanced Studies, New Delhi 110070, India; ORCID: <https://orcid.org/0000-0002-4410-566X>; Email: uditsoni.iitd@gmail.com


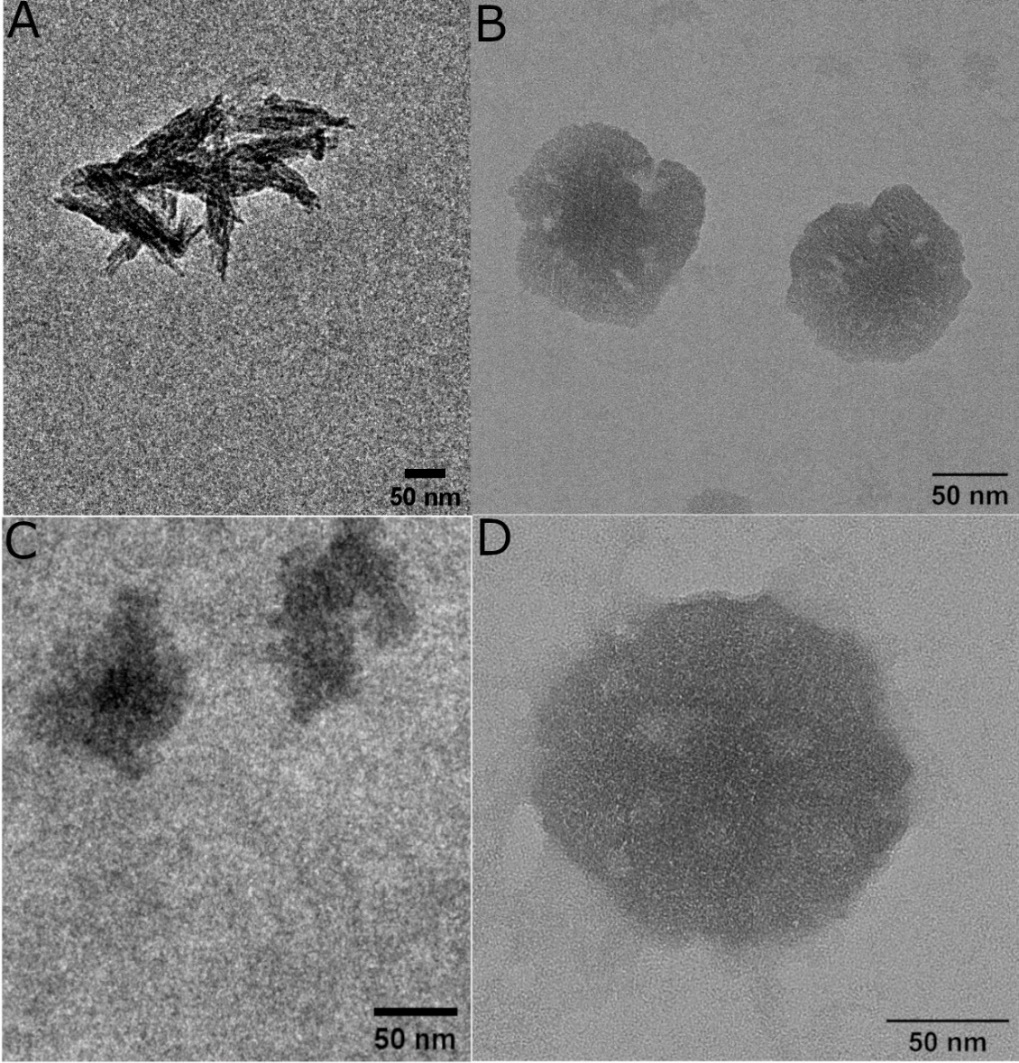


Supplementary Figure S1: Cryo-Transmission Electron Microscopy (cryo-TEM) images of the synthesized nanohybrids. A) Hydroxyapatite (HAP), B) Hydroxyapatite-urea (HAU), C) Magnesium doped hydroxyapatite-urea (MgHAU), and D) Zinc doped hydroxyapatite-urea (ZnHAU).


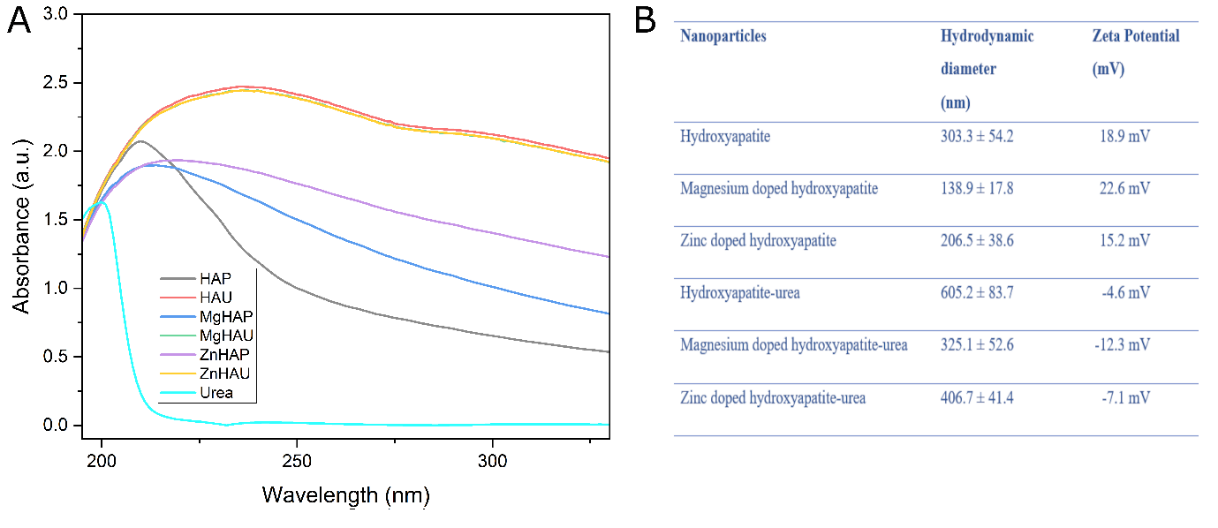


Supplementary Figure S2: **UV-visible spectrum of the synthesized urea-nanohybrids, bare nanoparticles, and urea.**


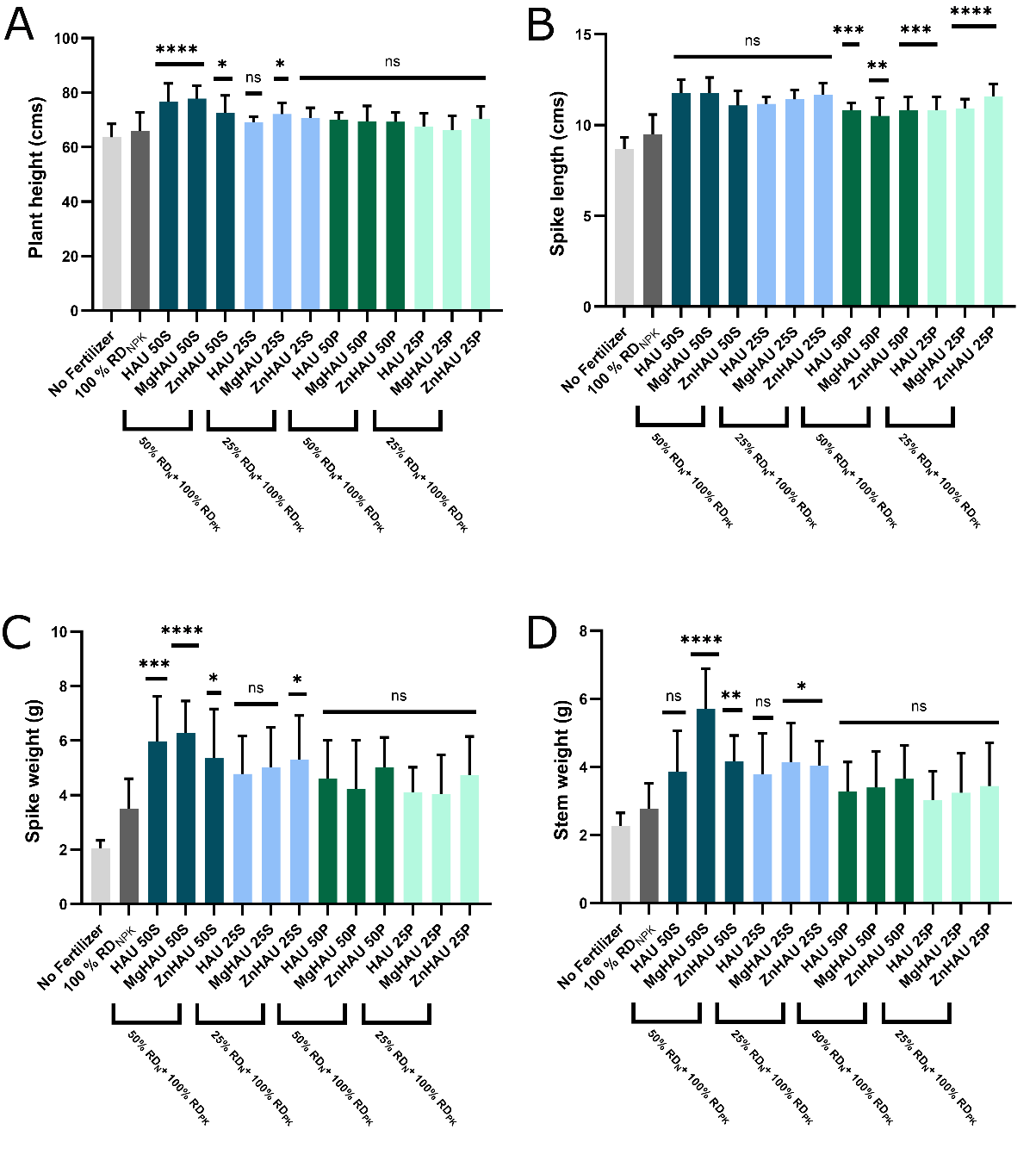


Supplementary Figure S3: Wheat growth and yield parameters: Plant height, spike length, spike weight, and stem weight of the harvested wheat plants. A) Plant height, B) Spike length, C) Spike weight, and D) Stem weight parameters of the wheat crop treated with a quarter and half dose of nitrogen as nanohybrids (suspension and pellet), full dose of nitrogen as urea (as control), and without fertilizer (No Fertilizer). All the nanohybrid treatments (HAU: Hydroxyapatite-urea, MgHAU: Mg-doped hydroxyapatite-urea, and ZnHAU: Zn-doped hydroxyapatite-urea) were compared with the control or 100% RD_NPK_ treatment. The values are provided as mean ± standard deviation, and statistical significance was calculated by one-way ANOVA with Dunnett’s multiple comparison test. The letters ‘*’, ‘**’, ‘***’, and ‘****’ represent ‘p < 0.05’, ‘p < 0.01’, ‘p < 0.001’, and ‘p < 0.0001’, respectively and ‘ns’ represents ‘not significant’.


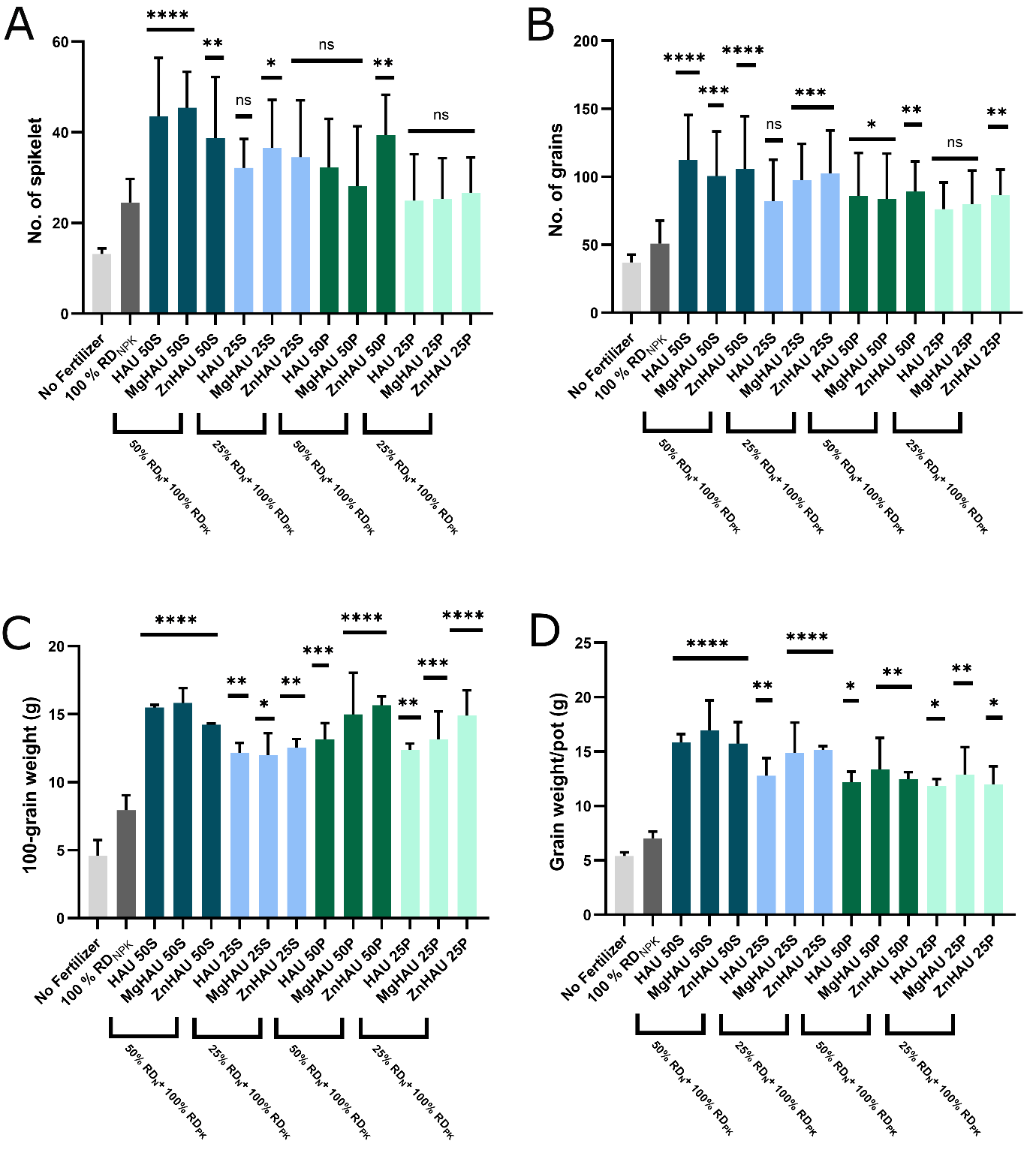


Supplementary Figure S4: Wheat growth and yield parameters: Number of spikelets, number of grains, 100-grain weight, and grain weight per pot of the harvested wheat plants. A) No. of spikelet, B) No. of grains, C) 100-grain weight, and D) Grain weight/pot parameters of the wheat crop treated with a quarter and half dose of nitrogen as nanohybrids (suspension and pellet), full dose of nitrogen as urea (as control), and without fertilizer (No Fertilizer). All the nanohybrid treatments (HAU: Hydroxyapatite-urea, MgHAU: Mg-doped hydroxyapatite-urea, and ZnHAU: Zn-doped hydroxyapatite-urea) were compared with the control or 100% RD_NPK_ treatment. The values are provided as mean ± standard deviation, and statistical significance was calculated by one-way ANOVA with Dunnett’s multiple comparison test. The letters ‘*’, ‘**’, ‘***’, and ‘****’ represent ‘p < 0.05’, ‘p < 0.01’, ‘p < 0.001’, and ‘p < 0.0001’, respectively and ‘ns’ represents ‘not significant’.

Supplementary Table S1: Hydrodynamic diameter (DLS) and Zeta potential measurements of measurements of synthesized urea-nanohybrids and bare nanoparticles.

| Nanoparticles |  | Hydrodynamic diameter (nm) | Zeta Potential (mV) |
| --- | --- | --- | --- |
| Hydroxyapatite |  | 303 ± 54.2 | 18.9 |
| Mg-doped hydroxyapatite |  | 138.9 ± 17.8 | 22.6 |
| Zn-doped hydroxyapatite |  | 206.5 ± 38.6 | 15.2 |
| Hydroxyapatite-urea |  | 605.2 ± 83.7 | -4.6 |
| Mg-doped hydroxyapatite-urea |  | 325.1 ± 52.6 | -12.3 |
| Zn-doped hydroxyapatite-urea |  | 406.7 ± 41.4 | -7.1 |

Supplementary Table S2: Macro-nutrients (Ca, Mg) composition in nanohybrids treated wheat plants. The levels of nutritional elements (Calcium, Magnesium) in the wheat stem, and root tissues after harvesting of the wheat crop treated with a quarter and half dose of nitrogen as nanohybrids (suspension and pellet), full dose of nitrogen as urea (as control), and without fertilizer (No Fertilizer). The values are provided as mean ± standard deviation, and statistical significance was calculated by one-way ANOVA with Dunnett’s multiple comparison test. The letters ‘*’, ‘**’, ‘***’, and ‘****’ represent ‘p < 0.05’, ‘p < 0.01’, ‘p < 0.001’, and ‘p < 0.0001’, respectively and ‘ns’ represents ‘not significant’.

| **Wheat macro-nutrients (Ca, Mg)** | | | | | | |
| --- | --- | --- | --- | --- | --- | --- |
| **Treatments** | **Calcium (mg/kg)** | | | **Magnesium (mg/kg)** | | |
|  | Grain | Stem | Roots | Grain | Stem | Roots |
| **No Fertilizer** | 207.22 ± 6.66 | 5455.55 ± 115.77 | 5622.22 ± 83.3 | 205.4 ± 4.4 | 935.8 ± 48.6 | 916.9 ± 6.7 |
| **Fertilizer (100 % RD_NPK_)** | 635.55 ± 13.09 | 10405.56 ± 168.53 | 8827.77 ± 79.4 | 323.2 ± 6.9 | 1956.8 ± 71.6 | 2089.4 ± 60.2 |
| **Hydroxyapatite-Urea 50% Suspension (50% RD_N_ + 100 % RD_PK_)** | 1135 ± 7.07  **** | 9566.66 ± 75  **** | 7622.22 ± 103.41  **** | 489.1 ± 7.5  **** | 1194.1 ± 51.4  **** | 2256.8 ± 39.8  ** |
| **Magnesium doped-Hydroxyapatite-Urea 50% Suspension (50% RD_N_ + 100 % RD_PK_)** | 1119.4 ± 42.16  **** | 9500 ± 111.80  **** | 8522.22 ± 44.0  **** | 656.2 ± 6.9  **** | 1888.4 ± 60.01  ns | 3377.7 ± 139.3  **** |
| **Zinc-Hydroxyapatite-Urea 50% Suspension (50% RD_N_ + 100 % RD_PK_)** | 1280 ± 7.5  **** | 8827.77 ± 75.4  **** | 9000 ± 93.5  **** | 346.2 ± 4.8  **** | 2352.2 ± 50.4  **** | 4034.8 ± 49.07  **** |
| **Hydroxyapatite-Urea 25% Suspension (25% RD_N_ + 100 % RD_PK_)** | 756.6 ± 6.12  **** | 7350 ± 79.0  **** | 7272.22 ± 66.6  **** | 427.02 ± 4.5  **** | 1748.3 ± 70.8  * | 2565.1 ± 49  **** |
| **Magnesium doped-Hydroxyapatite-Urea 25% Suspension (25% RD_N_ + 100 % RD_PK_)** | 745.55 ± 42.4  **** | 6822.22 ± 90.5  **** | 6972.22 ± 48.6  **** | 396.8 ± 8.5  **** | 1835.2 ± 206  ns | 2262.3 ± 48.4  ** |
| **Zinc-Hydroxyapatite-Urea 25% Suspension (25% RD_N_ + 100 % RD_PK_)** | 867.77 ± 19.22  **** | 7216.66 ± 150  **** | 6566.66 ± 61.2  **** | 435.2 ± 5.07  **** | 1866.6 ± 316  ns | 1906.6 ± 37.4  ** |
| **Hydroxyapatite-Urea 50% Pellet (50% RD_N_ + 100 % RD_PK_)** | 673.88 ± 21.90  *** | 10394.44 ± 130.9  ns | 8422.22 ± 66.6  **** | 397.7 ± 6.6  **** | 2126.08 ± 264  ns | 2291.3 ± 254  *** |
| **Magnesium doped-Hydroxyapatite-Urea 50% Pellet (50% RD_N_ + 100 % RD_PK_)** | 628.33 ± 13.69  ns | 8983.33 ± 79  **** | 9655.55 ± 95  **** | 484.4 ± 5.27  **** | 1462.08 ± 48.4  **** | 4642.3 ± 168.8  **** |
| **Zinc-Hydroxyapatite-Urea 50% Pellet (50% RD_N_ + 100 % RD_PK_)** | 748.88 ± 15.16  **** | 8672.22 ± 83.33  **** | 9355.55 ± 76.8  **** | 397.7 ± 6.6  **** | 1158.9 ± 49.5  **** | 2554 ± 51.1  **** |
| **Hydroxyapatite-Urea 25% Pellet (25% RD_N_ + 100 % RD_PK_)** | 769.44 ± 7.26  **** | 8350 ± 86.6  **** | 6994.44 ± 68.2  **** | 326.4 ± 4.8  ns | 1632.3 ± 251  *** | 1524.2 ± 46.3  **** |
| **Magnesium doped-Hydroxyapatite-Urea 25% Pellet (25% RD_N_ + 100 % RD_PK_)** | 749.44 ± 5.27  **** | 10800 ± 122.4  **** | 7250 ± 86.6  **** | 435.2 ± 5.07  **** | 1643.6 ± 51.8  *** | 1761.2 ± 111.1  **** |
| **Zinc-Hydroxyapatite-Urea 25% Pellet (25% RD_N_ + 100 % RD_PK_)** | 677.22 ± 14.38  *** | 7566.66 ± 75  **** | 7955.55 ± 63.4  **** | 364.9 ± 5.0  **** | 1356.9 ± 51.1  **** | 1252.2 ± 32.3  **** |

Supplementary Table S3: Micro-nutrients composition in nanohybrids treated wheat plants. The levels of nutritional elements (zinc, iron, and manganese) in the wheat stem, and root tissues after harvesting of the wheat crop treated with a quarter and half dose of nitrogen as nanohybrids (suspension and pellet), full dose of nitrogen as urea (as control), and without fertilizer (No Fertilizer). The values are provided as mean ± standard deviation, and statistical significance was calculated by one-way ANOVA with Dunnett’s multiple comparison test. The letters ‘*’, ‘**’, ‘***’, and ‘****’ represent ‘p < 0.05’, ‘p < 0.01’, ‘p < 0.001’, and ‘p < 0.0001’, respectively and ‘ns’ represents ‘not significant’.

| **Treatments** | **Zinc (mg/kg)** | | | **Iron (mg/kg)** | | | **Manganese (mg/kg)** | | |
| --- | --- | --- | --- | --- | --- | --- | --- | --- | --- |
|  | Grain | Stem | Roots | Grain | Stem | Roots | Grain | Stem | Roots |
| **No Fertilizer** | 5.99 ± 0.33 | 1.18 ± 0.07 | 2.28 ± 0.12 | 22.9 ± 0.23 | 332.35 ± 10 | 384.52 ± 2.5 | 16.2 ± 0.47 | 101 ± 3.3 | 61.4 ± 6.3 |
| **Fertilizer (100 % RD_NPK_)** | 13.20 ± 0.08 | 7.16 ± 0.05 | 14.01 ± 0.16 | 54.9 ± 1.4 | 578.8 ± 6.6 | 841.59 ± 5.2 | 40.3 ± 1.34 | 146.4 ± 4 | 101.1 ± 5 |
| **Hydroxyapatite-Urea 50% Suspension (50% RD_N_ + 100 % RD_PK_)** | 13.44 ± 0.34  ns | 6.29 ± 0.07  **** | 12.6 ± 0.23  **** | 68.1 ± 1.25  **** | 634 ± 1.8  **** | 795.9 ± 31.1  **** | 50.4 ± 1.08  **** | 126.8 ± 5.9  **** | 98.8 ± 7.6  ns |
| **Magnesium doped-Hydroxyapatite-Urea 50% Suspension (50% RD_N_ + 100 % RD_PK_)** | 15.28 ± 0.08  **** | 6.25 ± 0.05  **** | 10.7 ± 0.19  **** | 83.7 ± 4.2  **** | 532.5 ± 7  **** | 924.6 ± 8.23  **** | 68.6 ± 0.88  **** | 127 ± 5.3  **** | 125 ± 4.2  **** |
| **Zinc-Hydroxyapatite-Urea 50% Suspension (50% RD_N_ + 100 % RD_PK_)** | 18.57 ± 0.11  **** | 5.31 ± 0.09  **** | 18.65 ± 0.06  **** | 78.2 ± 1.1  **** | 582.9 ± 5.8  ns | 798.89 ± 7.5  **** | 57.8 ± 0.90  **** | 133.4 ± 4.6  **** | 85.6 ± 3.4  **** |
| **Hydroxyapatite-Urea 25% Suspension (25% RD_N_ + 100 % RD_PK_)** | 13.76 ± 0.17  **** | 4.76 ± 0.11  **** | 6.14 ± 0.16  **** | 52.6 ± 1.06  ns | 619.1 ± 5  **** | 935.23 ± 5.9  **** | 46.7 ± 0.78  **** | 130 ± 8.8  **** | 103 ± 4.2  ns |
| **Magnesium doped-Hydroxyapatite-Urea 25% Suspension (25% RD_N_ + 100 % RD_PK_)** | 14.36 ± 0.29  **** | 5.40 ± 0.09  **** | 11.6 ± 0.17  **** | 63.4 ± 0.35  **** | 518.1 ± 10  **** | 747.26 ± 4.5  **** | 48.4 ± 0.28  **** | 117.3 ± 3.3  **** | 106 ± 4.9  ns |
| **Zinc-Hydroxyapatite-Urea 25% Suspension (25% RD_N_ + 100 % RD_PK_)** | 13.68 ± 0.16  **** | 5.87 ± 0.07  **** | 7.19 ± 0.13  **** | 64.3 ± 0.62  **** | 553 ± 4.9  **** | 821.30 ± 6.7  ** | 50.5 ± 0.25  **** | 126.3 ± 5.4  **** | 110 ± 6.1  ** |
| **Hydroxyapatite-Urea 50% Pellet (50% RD_N_ + 100 % RD_PK_)** | 20.18 ± 0.09  **** | 3.41 ± 0.10  **** | 8.85 ± 0.08  **** | 104.3 ± 1.29  **** | 445.6 ± 5.5  **** | 523.23 ± 8.7  **** | 73.0 ± 0.98  **** | 134.57 ± 5.7  *** | 58.4 ± 4.5  **** |
| **Magnesium doped-Hydroxyapatite-Urea 50% Pellet (50% RD_N_ + 100 % RD_PK_)** | 21.74 ± 0.09  **** | 3.06 ± 0.27  **** | 4.83 ± 0.08  **** | 85.9 ± 4.1  **** | 512.6 ± 5  **** | 968 ± 8.3  **** | 69 ± 0.43  **** | 105.5 ± 5.5  **** | 121 ± 8.4  **** |
| **Zinc-Hydroxyapatite-Urea 50% Pellet (50% RD_N_ + 100 % RD_PK_)** | 24.53 ± 0.12  **** | 5.23 ± 0.07  **** | 9.91 ± 0.12  **** | 72.8 ± 1.26  **** | 545 ± 6.6  **** | 926.98 ± 4.6  **** | 74.8 ± 0.58  **** | 108.48 ± 5.3  **** | 132 ± 4.7  **** |
| **Hydroxyapatite-Urea 25% Pellet (25% RD_N_ + 100 % RD_PK_)** | 15.28 ± 0.09  **** | 4.17 ± 0.10  **** | 13.6 ± 0.13  *** | 53.1 ± 1.2  **** | 495.7 ± 4.3  ns | 481.2 ± 10.2  **** | 44.4 ± 0.93  **** | 128.44 ± 7.0  **** | 58.2 ± 4.6  **** |
| **Magnesium doped-Hydroxyapatite-Urea 25% Pellet (25% RD_N_ + 100 % RD_PK_)** | 16.69 ± 0.11  **** | 6.22 ± 0.08  **** | 4.73 ± 0.44  **** | 63.4 ± 1.11  **** | 627 ± 3.9  **** | 740.14 ± 4.7  **** | 47.1 ± 0.53  **** | 114.41 ± 4.9  **** | 85.5 ± 3.6  **** |
